# Search for the Function of NWC, Third Gene Within RAG Locus: Generation and Characterization of NWC-Deficient Mice

**DOI:** 10.1007/s00005-015-0379-1

**Published:** 2015-12-24

**Authors:** Monika Kasztura, Lukasz Sniezewski, Agnieszka Laszkiewicz, Michal Majkowski, Kamil Kobak, Karolina Peczek, Sylwia Janik, Violetta Kapusniak, Arkadiusz Miazek, Malgorzata Cebrat, Pawel Kisielow

**Affiliations:** Laboratory of Molecular and Cellular Immunology, Department of Tumor Immunology, L. Hirszfeld Institute of Immunology and Experimental Therapy, Polish Academy of Sciences, Wroclaw, Poland; Chair of Biostructure and Physiology, Department of Histology and Embryology, Wroclaw University of Environmental and Life Sciences, Wroclaw, Poland; Laboratory of Tumor Immunology, Department of Tumor Immunology, L. Hirszfeld Institute of Immunology and Experimental Therapy, Polish Academy of Sciences, Wroclaw, Poland

**Keywords:** Evolutionarily conserved genes, NWC-knockout mice, NWC protein function/partners, Recombination activating gene (RAG) locus, Cilia

## Abstract

*NWC* is a third gene within recombination activating gene (RAG) locus, which unlike RAG genes is ubiquitously expressed and encodes a unique protein containing three strongly evolutionarily conserved domains not found in any other known protein. To get insight into its function we identified several proteins co-immunoprecipitating with NWC protein and generated new NWC-deficient mice. Here, we present evidence that unlike many other ubiquitously expressed evolutionarily conserved proteins, functional inactivation of NWC does not cause any gross developmental, physiological or reproductive abnormalities and that under physiological conditions NWC may be involved in assembling and functioning of cilia, cell surface organelles found on nearly every eukaryotic cell.

## Introduction

*NWC* is a third evolutionarily conserved gene identified within recombination activating gene (RAG) locus (Cebrat et al. 2005). In contrast to lymphocyte specific RAG-1 and RAG-2 genes (Oettinger et al. 1990; Schatz et al. 1989), which encode V(D)J recombinase generating diversity of T and B cell antigen receptors, it is expressed in all cells except lymphocytes (Cebrat et al. 2005, 2008). The function of *NWC* is unknown. The predicted structure of vertebrate NWC protein contains three strongly conserved domains not found in any other proteins described in available databases. In vertebrates, these domains contain identical aminoacids at no less than 19 (65 %), 5 (83 %) and 14 (82 %) positions, respectively (Cebrat et al. 2005). The latter two domains are also very well conserved in many invertebrate species, including *Trichoplax adhaerens* (Placozoa) (Laszkiewicz et al. 2014). The overall identity of the whole NWC protein sequence in vertebrates is higher than 27 % (Cebrat et al. 2005; Laszkiewicz et al. 2014). Considering the evolutionary conservation, the unique structure of encoded protein and close association with RAG genes during vertebrate evolution, an effort to learn about the function of NWC gene and protein seems to be well justified. For this purpose, we generated *NWC*-deficient mice and attempted to find proteins binding to NWC protein. Here, we identify the candidate NWC partner proteins and report that *NWC*-deficient mice are viable, fertile and show no obvious developmental, anatomical and functional defects.

## Materials and Methods

### Animals

All procedures using animals were reviewed and approved by First Local Ethical Commission for Animal Experimentation in Wroclaw at the Institute of Immunology and Experimental Therapy (permit number 13/2009). Mice were killed under sodium thiopental anesthesia.

### Genetically Modified Mice

NWC^tmproI^ and reporter RAG-2-GFP/NWC-YFP (BAC-RG/NY) mice were obtained as described (Laszkiewicz et al. 2011, 2014). The reporter BAC-RG/ΔpNY mice were obtained by deletion of the NWC promoter region (–151/+537) nucleotides relative to NWC transcriptional start site from the original RAG-2-GFP/NWC-YFP BAC by ET recombination as described (Laszkiewicz et al. 2014). The RG/ΔpNY BAC was used to generate transgenic C57BL/6 mice strains at Karolinska Center for Transgene Technologies, Karolinska Institute, Stockholm. B230118H07Rik^tm1a(KOMP)Wtsi^ (NWC-KOMP) and B6.C-Tg(CMV-cre)1Cgn/J (Cre-deleter) mice were purchased from Jackson Laboratory, UC Davis. The observations reported in the present paper were made on the group of WT and homozygous NWC-KOMPcre mice. In the offspring of mated heterozygous NWC-KOMPcre mice we found all three genotypes; WT, WT/NWC-KOMPcre and NWC-KOMPcre/NWC-KOMPcre.

### Genotyping

Genomic DNA was isolated from tail fragment of 6-week-old mice by proteinase K (Roche, Switzerland) digestion followed by phenol/chloroform/isoamyl alcohol extraction and ethanol precipitation.

NWC-KOMP: polymerase chain reactions (PCR) were performed using primer pair KOzygF (5′–AAGGGTATGTGCTCTCCTTC, forward)/KOzygR (5′–CAATTTGTAAGACAGTTCTG, reverse). Touch-down PCR temperature profiles were used: an initial denaturation step at 94 °C followed by ten cycles of denaturation steps at 94 °C for 30 s, primer annealing with 1 °C temperature drop per cycle starting from 65 °C for 30 s, extension at 72 °C for 30 s followed by 30 cycles of denaturation steps at 94 °C for 30 s, primer annealing at 55 °C for 30 s and extension at 72 °C for 30 s.

Offspring of homozygous NWC-KOMP × cre-deleter: WT, KOMP and LoxP/Cre recombined, exon5-less NWC (NWC-KOMPcre) alleles were distinguished in a single PCR with primers: KOzygF, KOzygR and Cre5KOF (5′-GCGTCGAGAAGTTCCTATTC, forward). Presence of the Cre recombinase gene was determined by a PCR using oIMR1084 (5′-GCGGTCTGGCAGTAAAAACTATC, forward) and oIMR1085 (5′-GTGAAACAGCATTGCTGTCACTT, reverse) primers and a PCR consisting of an initial denaturation step at 94 °C followed by 30 cycles of denaturation steps at 94 °C for 30 s, primer annealing at 55 °C for 30 s and extension at 72 °C for 30 s. The progeny of NWC-KOMP and cre-deleter matings was intercrossed to remove Cre transgene from homozygotes bearing LoxP/Cre recombined NWC allele.

### RT-PCR

RNA was extracted from homogenized tissues using Tri Reagent (Molecular Research Center, Inc., USA) according to the manufacturer’s instructions. Five microgram of extracted RNA was reverse-transcribed with Superscript III reverse transcriptase (Invitrogen, USA) with 200 ng of random hexamers. cDNA was amplified for 30 cycles using an annealing temperature of 55 °C (for Hprt) or 57 °C (for NWC). The following primer pairs were used: HPRT: HprtF GCTGGTGAAAAGGACCTCT, HprtR CACAGGACTAGAACACCTGC; NWC exons 2–4: NWCexIIF CTCAAATACCGAGGCCAGAG, NWCexIVR CACCTTGAAAGGAACTCCCA; NWC exons 3–6: NWC 21R GGGAGCACATGGAACTGAT, NWCTagR CCTCAGAGGTGAGCGGTAGGT. To control for genomic DNA contamination, an equal amount of total RNA was amplified without previous reverse transcription (RT: control). PCR products obtained by amplifying serially diluted cDNA were separated by agarose-gel electrophoresis and visualized with Midori Green Advance staining (Nippon Genetics Europe GmbH, Germany).

### NWC Specific Antibody (Ab285)

Rabbit (Flemish giant) was immunized subcutaneously and intramuscularly three times with the recombinant GST-tagged NWC fragment (123–180 a–a) (100 µg per injection) emulsified in S-6322 adjuvant (Sigma-Aldrich, USA) and bled 14 days after last injection. Serum was affinity purified on recombinant NWC-His tagged protein immobilized on CNBr Sepharose (Amersham, UK) and resulting polyclonal antibody (Ab285) was stored as 50 % glycerol stocks at 4 °C.

### Western Blotting

Tissues were mechanically disrupted using a Polytron homogenizer (Glen Mills Inc., USA) and lysed on ice in 1 % Nonidet P-40 in buffer (50 mM Hepes, pH 7.2, 150 nM NaCl, 0.5 % sodium deoxycholate, 0.1 % SDS, 1× complete protease inhibitors (Roche, Switzerland) for 30 min followed by sonication. Insoluble fragments were removed by centrifugation (20,000*g*, 20 min). Protein concentrations were measured by BCA (Pierce, Rockford, IL). Clear lysates (20–50 µg) were separated on 4–20 % polyacrylamide gels and electro-transferred to PVDF membranes (Millipore, USA) in Towbin buffer (25 mM Tris, 192 mM glycine) supplemented with 10 % methanol. The membranes were blocked in 10 % skimmed milk overnight and incubated with primary antibody [Ab285 or anti-actin (C-11, Santa Cruz Biotechnolgy, USA)] for 60 min. After washing the membranes were incubated for 30 min with secondary horseradish-conjugated, anti-rabbit IgG or anti-goat antibody (Santa Cruz Biotechnology, USA) and developed by enhanced chemiluminescence detection system (Roche, Switzerland).

### Flow Cytometry

Flow cytometry was performed on FACS-Aria and FACS-Calibur machines (Beckton-Dickinson, USA). Cell suspensions were prepared as follows:

Organs of wild-type, BAC-RG/NY and BAC-RG/ΔpNY mice were washed in ice-cold phosphate-buffered saline (PBS) to remove blood, cut into small 1–2 mm^2^ pieces and incubated in enzymatic solutions at 37 °C for various time-points: (1) lungs: 2 mg/ml Collagenase type 2 and 150 μg/ml DNAse I—1 h; (2) heart 1 mg/ml Collagenase type 2 and 10 μg/ml DNAse I—45 min; (3) kidney: 1 mg/ml Collagenase type 1—40 min; (4) liver: 0.5 mg/ml Collagenase type 1—30 min, skeletal muscle: 2 mg/ml Collagenase type 2–30 min, 1 mg/ml Papain—25 min; (5) brain: 1 mg/ml Collagenase type 1 and 10 μg/ml DNAse I—10 min. Tissue pieces were centrifuged (1200 rcf, 5 min, 4 °C), resuspended in 10 μg/ml DNAse I solution and incubated at room temperature for 15 min. The solution was mixed every 5 min. For testes only second step of enzymatic digestion was performed. Lymph nodes, spleen and thymus were isolated, washed in ice-cold PBS and strained through 70-μm mesh. Bone marrow cells were isolated by washing the femur and tibia with ice-cold PBS stream. All tissues and cells were mechanically dissociated by syringe trituration, washed twice with PBS by centrifugation (1200 rcf, 5 min, 4 °C) and resuspended in PBS [5 % fetal bovine serum (FBS)]. Single cell suspension was filtered through 70-μm mesh.

### Immunohistochemistry

Testes obtained from wild-type and NWC-KOMPcre mice were fixed with formalin and paraffin-embedded. The paraffin sections were cut at 4 µm thickness using microtome. The sections were stained with hematoxylin and eosin, dehydrated in ascending grades of alcohol, cleared in xylene, mounted with DPX and used for histomorphological analysis.

Immunohistochemical analysis was performed on paraffin-embedded sections of testis fixed with 4 % formalin. Paraffin sections were rehydrated, immersed in citrate buffer (pH 6.0) at 97 °C for 20 min and blocked with 20 % normal goat serum (NGS) in PBS for 1  h. An avidin/biotin (Vector Laboratories Inc., Burlingame, CA, USA) blocking step with 3 % hydrogen peroxide was included before the addition of the primary antibody. Incubations with primary Ab285 antibody (diluted 1:200 with NGS in PBS) or anti-PCNA antibodies (sc-7907, Santa Cruz Biotechnology, USA) (diluted 1:100) were performed overnight at room temperature in humid chambers. Negative controls were incubated without primary antibodies. The EnVision™ Systems (Dako) detection system was used, and 3,3′‐diaminobenzidine served as the chromogen. Slides were counterstained with Mayer’s hematoxylin, than examined and photographed under a light microscope (Nikon Eclipse 80i; Nikon, Melville, NY, USA).

### Immunoprecipitation and Co-immunoprecipitation

Testes were homogenized using Dounce homogenizer in 0.2 % Tween-20 (50 mM Tris, pH 7.4, 150 nm NaCl) containing leupeptin (1 µg/ml), aprotinin (1 µg/ml), pepstatin (1 µg/ml) and PMSF (1 mM). The homogenate was incubated on ice for 30 min, centrifuged for 15 min (20,000*g*) at 4 °C to remove cellular debris. Lysates were incubated for 1 h on Protein G resin without antibodies to remove unspecifically interacting proteins. Cleared samples were then incubated overnight at 4 °C with 15 μg of anti-NWC rabbit polyclonal antibody Ab285 or control rabbit IgG immobilized on Protein G. Beads were washed three times in (1 ml each) with lysis buffer and eluted with 0.1 M glycine at 25 °C for 15 min. Eluents were titrated with neutralization buffer (1 M Tris pH 8.8; 1/5 of the elution volume) to physiological pH, boiled in 2× loading buffer for 5 min and separated on 4–20 % polyacrylamide gels (BioRad, Hercules, CA). Proteins were silver-stained, band corresponding to immunoprecipitated NWC protein was cut from gel and analyzed by liquid chromatography coupled to tandem mass spectrometry (LC–MS–MS/MS).

Co-immunoprecipitation (Co-IP) was performed with the Thermo Scientific™ Pierce™ Co-Immunoprecipitation Kit enabling isolation of native NWC-protein partner complexes from a testes lysates by direct immobilization of purified Ab285 antibody onto an agarose support. Columns with antibodies, testes lysates and all solutions for Co-IP experiments were prepared using manufacturer buffers. Briefly, testes from wild-type and NWC-KOMPcre mice were homogenized using Dounce homogenizer in IP lysis buffer, incubated on ice for 30 min, centrifuged for 15 min (20,000*g*) at 4 °C to remove cellular debris and clarified for 1 h on a resin without antibodies. Cleared lysates were incubated overnight at 4 °C with 15 μg of immobilized on Co-IP resin with Ab285 antibody or control rabbit IgG. Beads were washed three times with the washing buffer and eluted with 100 µl of the elution buffer. Eluted proteins were analyzed on silver stained SDS-PAGE gel, by Western blotting and finally by LC–MS–MS/MS.

### Lc–MS–MS/MS

Peptides mixtures were analyzed by LC–MS–MS/MS using Nano-Acquity (Waters, USA) LC system and Orbitrap Velos mass spectrometer (Thermo Electron Corp., USA) in Laboratory of Mass Spectrometry, Institute of Biochemistry and Biophysics, Warsaw, Poland. Raw data were processed by Mascot Distiller followed by Mascot Search (Matrix Science, UK, on-site license) against Swiss-Prot database (20140124). Peptides with Mascot Score exceeding the threshold value corresponding to <5 % false positive rate, calculated by Mascot procedure, and with the Mascot score above 30 were considered to be positively identified.

### Immunofluorescence and Imaging

NIH-3T3 cells (cell line collection, IIET PAS) were grown in DMEM (4.5 % of glucose) containing 10 % FBS, penicillin (100 U/ml), streptomycin (0.1 mg/ml), l-Glutamine (2 mM), β-mercaptoethanol (9 µM). For immunofluorescence cells were grown to 90–100 % confluency on sterile coverslips placed in 24-well plate. Cells were cultured in serum-free medium for 24 h before fixation to promote cilia formation (Pitaval et al. 2013). Cells were fixed in 3.7 % formalin in PBS and incubated for 10 min at room temperature, permeabilized with 0.1 % Triton X-100 in PBS (10 min), washed with PBS and blocked for 1 h with PBS containing 1 % FBS. Cells were then incubated for 1 h at RT with primary (Ab285: 1/100; mouse monoclonal against acetylated α-tubulin: 2 μg/ml) and secondary (Cy2 conjugated donkey anti-rabbit: 15 µg/ml, Cy5 conjugated goat anti-mouse: 15 µg/ml) antibodies, respectively. Control reactions were carried out without primary antibodies. Labeled cells were mounted in moviol based mounting medium containing DAPI (2 μg/ml) as a nuclei marker. Cells were imaged by spinning disc confocal microscope Zeiss Cell Observer SD equipped with Yokogawa CSU-X1A 5000 unit with the use of 63 × oil-immersed Plan-Apochromat objective (NA 1.4). Z-stacks across entire cells were acquired at Z increments of 0.4 μm. Images were processed with the use of ImageJ (NIH).

## Results

### Generation and Characterization of NWC-Deficient Mice Lacking NWC Protein Containing Evolutionarily Conserved Domains

**Fig. 1:**
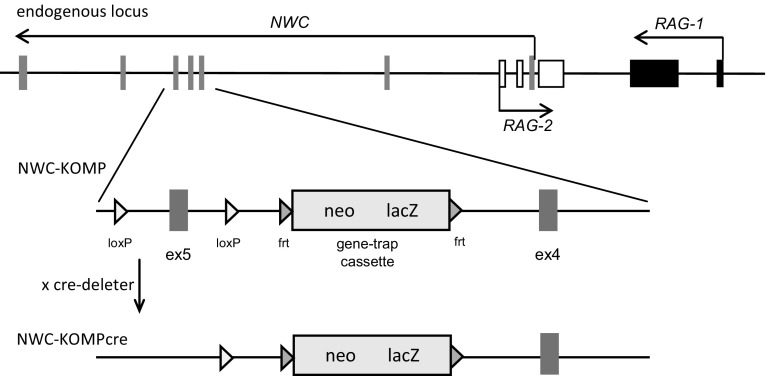
The schematic representation of endogenous RAG/NWC locus and its modifications in NWC-KOMP and NWC-KOMPcre mice. NWC-KOMPcre mice were obtained by crossing NWC-KOMP mice with mice expressing cre-recombinase. The relative positions of exons encoding RAG-1 (*black boxes*), RAG-2 (*open boxes*), and NWC (*gray boxes*) are shown. Horizontal arrows indicate transcription start sites and orientations

**Fig. 2:**
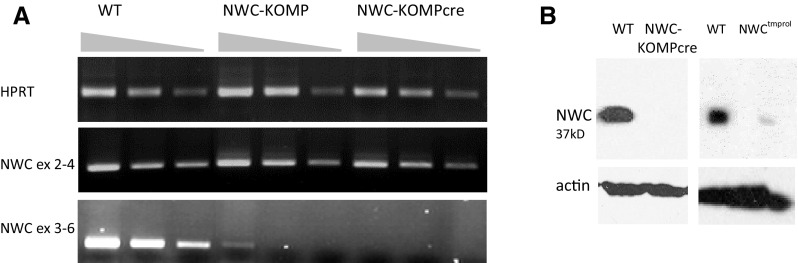
NWC expression in testes of the wild-type vs NWC-deficient mice: **a** RT-PCR, **b** Western blotting. Primers amplifying the regions of the transcript localized upstream (exons 2 and 4) and downstream (exons 3 and 6) of the termination cassette were used in RT-PCR. *Triangles* indicate decreasing concentration of the cDNA. Western blotting was performed using NWC specific Ab285 antibody. The difference between predicted (~28 kDa) and observed (~37 kDa) molecular weight of NWC protein is due to its high negative charge

As previously reported, strong but not complete inhibition of *NWC* transcription in *NWC*-deficient NWC^tmpro1^ mice, resulting from the deletion of the first and main *NWC* promoter, had no apparent phenotypic effect (Laszkiewicz et al. 2011). We reasoned that the lack of detectable change of the phenotype could be due to the residual *NWC* transcription of normal, non-mutated gene, regulated by the secondary *NWC* promoter that we discovered outside of the deleted region (Laszkiewicz et al. 2011). Therefore, to test this possibility we attempted to generate NWC-deficient mice unable to produce functional NWC protein by taking advantage of heterozygous B230118H07Rik^tm1a(KOMP)Wtsi^ (NWC-KOMP) mice. These mice contain a gene trap cassette inserted in *NWC* intron 4, and exon 5 flanked by loxP sequences (Fig. 1) preventing generation of the transcript encoding functional NWC protein with conserved domains. The heterozygous NWC-KOMP mice were intercrossed and expression of *NWC* in homozygous progeny was analyzed by RT-PCR, which showed that the synthesis of full-length *NWC* transcript was strongly suppressed but not completely abrogated (Fig. 2a, middle panel). We therefore crossed homozygote NWC-KOMP mice with B6.C-Tg(CMV-cre)1Cgn/J (cre-deleter) mice expressing cre-recombinase under the control of CMV promoter in order to delete the exon 5 of NWC gene. The homozygous NWC-KOMPcre progeny lacked detectable expression of the full-length *NWC* transcript (Fig. 2a, right panel) and NWC protein (Fig. 2b, left panel). Expression of NWC protein was analyzed by Western blotting using affinity purified polyclonal NWC specific antibody (Ab285). The NWC specificity of Ab285 antibody is indicated by the fact that in wild type mice it detected a single 37 kDa protein band, which in NWC^tmpro1^ mutant mice, characterized by strong inhibition of *NWC* transcription (Laszkiewicz et al. 2011), was hardly detectable (Fig. 2b, right panel). In the Ab285 immunoprecipitate, the NWC protein was detected by MALDI-TOF mass spectrometry (not shown), confirming the identity of recognized protein and providing definitive evidence that NWC protein is expressed in normal cells. The homozygous NWC-KOMPcre mice were observed for 6 month and showed no obvious morphological, anatomical, physiological or reproductive abnormalities. However, the possibility that transcription of the remaining exons 1–4 could result in the synthesis of truncated NWC protein, which is not recognized by Ab285 antibody, cannot be excluded. Therefore, we cannot completely rule out that such protein, if present, could be responsible for the lack of the phenotype of NWC-KOMPcre mice. Yet, this possibility is highly unlikely because the lack of the evolutionarily conserved domains encoded by exons 5–7, in all probability would render NWC protein non-functional.

To look for possible subtle effects of the introduced modification we decided to analyze in more detail the tissue that in normal mice express the highest level of NWC protein.

### Tissue Expression Level of NWC Protein

**Fig. 3:**
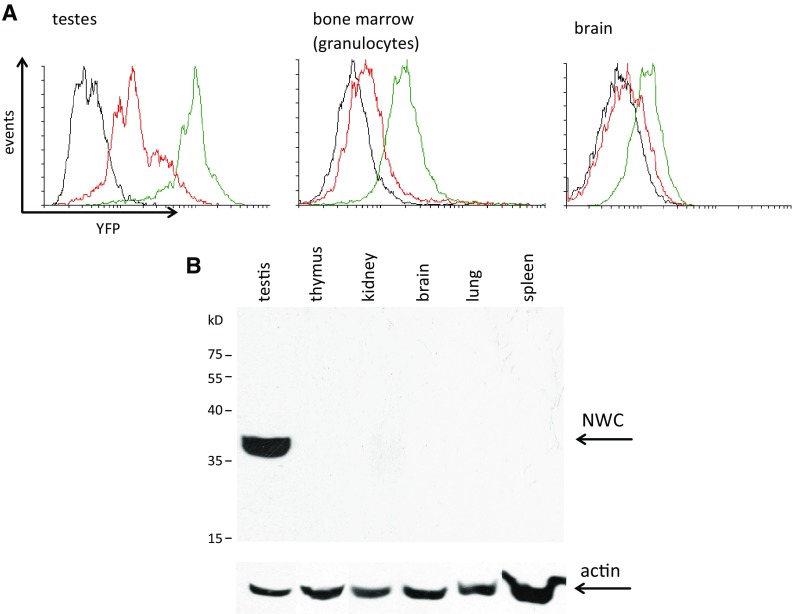
Tissue expression of NWC protein. **a** Expression of NWC/YFP fusion protein—assayed by flow cytometry—in cells from indicated tissues from BAC-RG/NY (*green*), BAC-RG/DpNY (*red lines*) and wild-type mice (negative control, *black lines*). **b** Expression of NWC protein in indicated tissues of normal mice, detected by western blotting with Ab285 antibody

In order to determine the level of NWC protein in different tissues we initially used BAC-RG/NY reporter mice harboring BAC-based transgene containing complete murine *RAG/NWC* locus. The transgene encoded green fluorescent GFP/RAG-2 fusion protein under the control of RAG-2 promoter and yellow fluorescent NWC/YFP fusion protein (Laszkiewicz et al. 2014). BAC-RG/NY mice with deleted *NWC* promoter region (BAC-RG/ΔpNY) and wild-type mice served as a control. As shown in Fig. 3a, high expression level of the reporter NWC/YFP protein was detected in testes and lower level in brain and bone marrow consistent with the expression level of NWC transcript (Laszkiewicz et al. 2012). In other tissues NWC/YFP fusion protein was undetectable (not shown). The results obtained in BAC reporter mice indicating that testes express the highest level of NWC protein were confirmed by Western blotting experiments using tissues from normal mice (Fig. 3b). Expression of NWC protein could be easily detected in testes, while in other organs it was difficult to detect by this method and required much higher concentration of protein (not shown). These results are in line with observation, that the expression level of NWC transcript is much higher in testes than in other tissues (Laszkiewicz et al. 2012). Previously (Kasztura et al. 2009), using another antibody (MoM12) reacting with recombinant NWC protein, we detected similar level of expression of recognized protein in all tissues examined, but recently we have found that the MoM12 may react with another protein of similar molecular weight, raising doubts concerning the interpretation of this earlier result.

### Comparison of Tissue Architecture of Testes of Normal and NWC-Deficient Mice

**Fig. 4:**
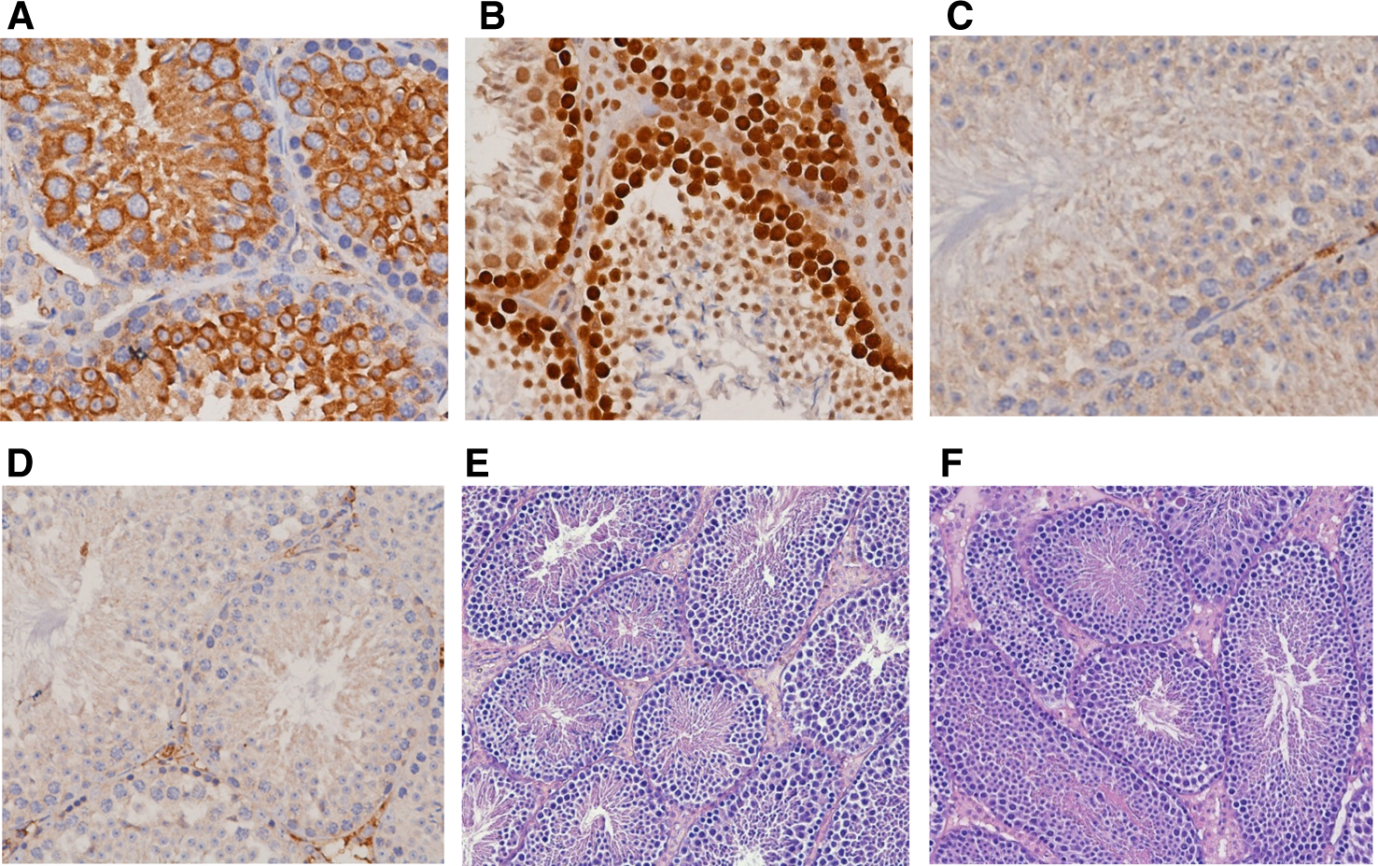
Formalin-fixed, paraffin-embedded wild-type (**a**, **b**, **c**, **e**) and NWC-KOMPcre (**d**, **f**) murine testes stained with: NWC specific Ab285 antibody (**a**, **d**), anti-PCNA antibody (**b**) and secondary reagent alone (**c**) followed by hematoxylin staining (**a**–**d**) or hematoxylin/eosin alone (**e**, **f**)

On the basis of above results, testes were chosen as a tissue for further analysis. The immuno-histological staining of tissue sections revealed that NWC protein is localized predominantly in cytoplasm of the cells in the germinal layer in wild-type mice (Fig. 4a) as opposed to nuclear localization of PCNA antigen which was used as control (Fig. 4b). As expected, no signal was observed in the testes of NWC-KOMPcre mice (Fig. 4d). The comparison of tissue architecture of testes from normal and *NWC*-deficient mice revealed no difference in tissue organization, cellularity or morphology (Fig. 4e, f).

### Identification of Candidate NWC Binding Proteins

**Fig. 5:**
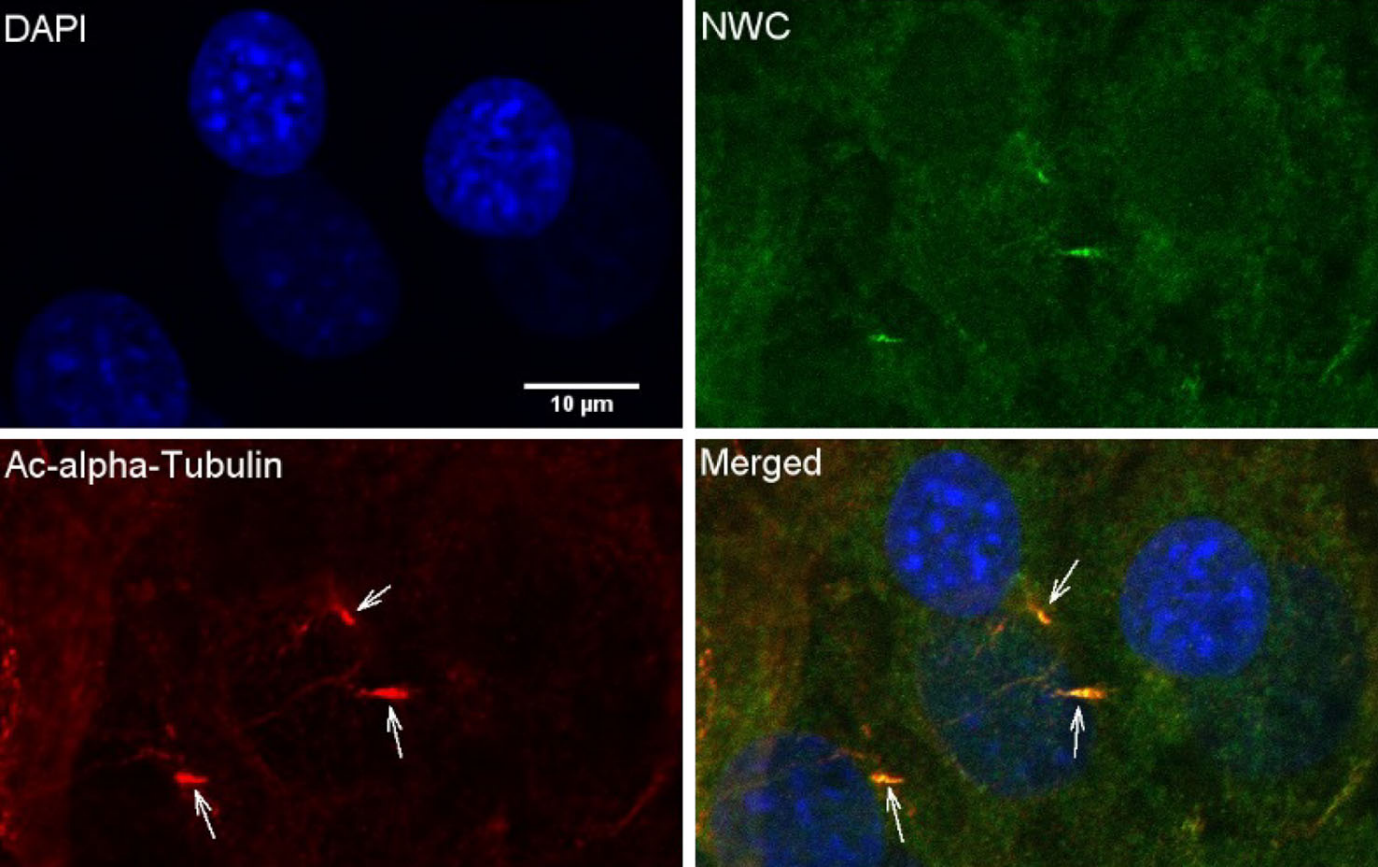
Co-localization of NWC protein with cilia. After inducing ciliogenesis NIH3T3 cells were immunostained with anti-NWC Ab285 antibody (*green*), anti-acetylated alpha-tubulin antibodies (*red*) and DAPI (*blue*) and analysed using confocal microscopy imaging. The merged image shows co-localization of NWC and acetylated alpha-tubulin in cilia projecting from the cell surface (*white* *arrows*)

The failure to observe any visible effects on the phenotype of NWC-KOMPcre mice prompted us to use an indirect approach to get insight into the function of *NWC* by searching for the protein partners binding to the NWC protein. To identify proteins interacting with NWC protein we performed co-immunoprecipitation experiments. The proteins co-precipitated with Ab285 antibody from lysates of testes from normal mice were identified by mass spectrometry. Protein lysates from testes of NWC-KOMPcre mice served as a negative control. Four independent experiments were performed. Eight proteins, specifically and repeatedly precipitated from tissue lysates of normal mice, represent the possible candidates of NWC binding proteins. They are listed in Table 1. Several of these proteins are known to be involved in the formation and functioning of the cilia. To support these findings we have checked whether NWC protein is present in those cell structures. To do this we have induced ciliogenesis in NIH3T3 cells by culturing in serum-free medium and stained with Ab285 antibody and anti-acetylated alpha tubulin (cilia marker). The results showed that NWC is localized in most of the primary cilia projecting from the cell surface (Fig. 5).

**Table 1:** Proteins co-immunoprecipitated with NWC protein, identified in LC–MS–MS/MS analysis

Proteins	Accession number	Wild-type mouse	NWC-KOMPcre mouse
Carbonic anhydrase 2	P00920	193.1 (±78.4)	ND
Dynein light chain roadblock-type 1	P62627	108.4 (±28.5)	ND
IFT-139 (Ttc21b)	Q0HA38	3889.7 (±863.6)	ND
IFT-144	Q3UGF1	229.9 (±128.6)	ND
Radial spoke head protein 6 homolog A	Q8CDR2	177.1 (±67.1)	ND
Radial spoke head protein 9 homolog	Q9D9V4	263.8 (±136.4)	ND
IFT-122	Q6NWV3	3850.6 (±1291.2)	332.0
IFT-43	Q9DA69	729.8 (±122.4)	ND

Numbers indicate the means of Mascot scores and SD obtained in four experiments

*ND* not detected

## Discussion

In the present work we showed that inability to synthesize *NWC* protein with conserved domains did not result in embryonic lethality or in any gross anatomical or physiological abnormalities in young mice. Given the extent of the evolutionary conservation and expression of *NWC* transcript in virtually all tissue types examined, one could have expected that the encoded protein, like many other evolutionarily conserved genes and proteins (Amsterdam et al. 2004; Jordan et al. 2002; Liao and Zhang 2007; White et al. 2013) will play essential cellular functions, and that the deletion of exons encoding conserved domains will result in severe developmental defects. From this point of view the lack of any obvious influence on the phenotype of *NWC*-deficient mice may seem surprising but is in line with other evidence (Ahituv et al. 2007) challenging the long prevailing notion that conserved elements, which show little variation across species, necessarily have essential functions. It is possible, however, that NWC has been playing essential role in evolution but this could become evident only outside the controlled laboratory environment, on a longer timescale over several generations (Koonin 2009).

Since the inactivation of *NWC* in NWC-KOMPcre mice did not give any clue to the function, we attempted to identify proteins binding to NWC protein and in this indirect way obtain information about its possible physiological role. Interestingly, seven out of eight identified proteins are involved in the intraflagellar transport (IFT)—IFT-122, IFT-43, IFT-144, tetratricopeptide repeat (TPR) protein 21b and dynein light chain roadblock-type 1 proteins. The intraflagellar transport is responsible for the development and maintenance of the cilia by coordinating rapid and bidirectional transport between the cell body and the tip of the cilium (Hao and Scholey 2009). Cilia themselves are specialized cell structures projecting from the cell surface of most of the eukaryotic cells and are involved in cell motility (motile cilia), signalling and sensory reception. Cilia are assembled by IFT in which multimeric protein complexes consisting of several IFT proteins and their cargo are transported bidirectionally along the axoneme with the use of IFT motors (kinesins and dyneins). Among the identified group of proteins, IFT-122, IFT-43, IFT-144 and TPR repeat 21b proteins are components of IFT-A complex involved in the retrograde transport, i.e. from the cilia tip towards the cell body. It is also worth noting that radial spoke head 9, radial spoke head 6a proteins are structural components of the sperm cilium (flagellum) (Satir and Christense 2007), providing possible explanation for the high expression of *NWC* in testes. Our demonstration that NWC co-localizes with primary cilia induced on the surface of NIH3T3 cells supports the results of co-precipitation experiments suggesting that NWC belongs to the IFT complex. Moreover, it is important to note that IFT-43, IFT-122 and IFT-144 proteins were also found to interact with human homolog of NWC protein, encoded by c11orf74 gene (Chatr-Aryamontri et al. 2015; Huttlin et al. 2015). This experiment, being a part of a high-throughput proteomic mapping approach, was performed by overexpression of affinity capture of HA-tagged human NWC protein in HEK293T cells and subsequent identification of the associated interaction partners by mass spectrometry. The fact that the same proteins were identified as NWC partners in different type of cells, species and using different approach strengthens the likelihood that they interact with NWC under physiological conditions. Further experiments are needed to investigate the exact role of NWC protein in the intra-flagellar transport and function of cilia.
